# Supplementary material for: Bioinformatics profiling identifies seven immune-related risk signatures for hepatocellular carcinoma
Source: PeerJ. 2020 May 26;8:e8301. doi: 10.7717/peerj.8301 (PMC7258897; doi:10.7717/peerj.8301)
Supplement: Table S1 [file peerj-08-8301-s001.docx]

**Table.1 Primers for RT-qPCR**

| Genes | Forward (5'-3') |  | Resvers (5'-3') |
| --- | --- | --- | --- |
| CFHR1  APOA2 | GGGGAGAAGCAACATTTTGTGA  CTGATGGAGAAGGTCAAGAGC |  | ACAGGAAGTGTCAGTGGACCT  GCTGTGTTCCAAGTTCCATG |
| IL-27 | CCTGATGTTTCCCTGACCTTC |  | GCTGCATCCTCTCCATGTTG |
| FCGRT  CD1D  CTSE  NCOA6  GAPDH | CACCTTCCTGCTATTCTCCTG  CAGGGAAGTCGGAACTGAAG  GGACTTGCCGATGTTTTCTG  TCAGAACCGAAGCCAAGATG  AGGTCGGTGTGAACGGATTTG |  | AGGTAAGCACGGAAAAGCC  GCATCCACTTCACCCATACAG  GAGAAATGGGAGTGGTCGTAG  GGGTATCATAACAGTGGCAGG  GGGGTCGTTGATGGCAACA |
